# Monitoring Vital Signs and Movement Using Contactless Sensors: Building a Home of the Future

**DOI:** 10.1093/geroni/igaf122.1769

**Published:** 2025-12-31

**Authors:** Elinor Schoenfield, Elinor Schoenfeld, Mengjing Liu, Isac Park, Suvab Baral, Tristan Vozzolo, Jason Mathew, Fan Ye

**Affiliations:** Stony Brook University Renaissance School of Medicine, Stony Brook, New York, United States; Renaissance School of Medicine at Stony Brook University, Stony Brook, New York, United States; Stony Brook University, Stony Brook, New York, United States; Stony Brook University, Stony Brook, New York, United States; Stony Brook University, Stony Brook, New York, United States; Stony Brook University, Stony Brook, New York, United States; Stony Brook University, Stony Brook, New York, United States; Stony Brook University, Stony Brook, New York, United States

## Abstract

As Americans live longer, there is a growing need to develop and implement technologies to monitor changes in health, supporting aging in place. We have installed privacy-preserving ultra-wideband wireless sensors in a model home to facilitate sensor and algorithm development for future home deployment. These sensors monitor vital signs and provide localization/tracking and activity recognition. We have developed a protocol to continuously collect and measure activities of daily living, location, and gait. We developed a multi-modal app using videos, audio, and texts to guide individuals and track activity completion time. To protect data privacy, raw sensor data is sent to a research cloud, and identifiable image data is sent to a university-supported HIPAA-compliant Box app. We spent >10 months developing, evaluating, stress testing, and improving our system and cloud infrastructure stability to ensure <1% data loss. We completed 19 sessions with volunteers to test our app and protocol, provide feedback, and continue to update our procedures and app before beginning data collection with individuals post-stroke. Recovery progress in post-stroke study participants will complete 3 study visits over 14 weeks to capture clinical and sensor measurements. We continue to refine our home deployment protocols for older adults with diverse technological knowledge. This presentation highlights our progress in developing and deploying our home monitoring system. We will discuss the challenges and procedures to collect, analyze, and interpret continuous physiologic and movement data in real-time, providing information, knowledge, and insights that will become vital to supporting the health of our expanding aging population.

